# Consumer segmentation and drivers of egg purchasing behavior among emerging consumers in central Chile: The role of nutritional knowledge, production systems, and quality perception

**DOI:** 10.1016/j.psj.2026.107161

**Published:** 2026-05-22

**Authors:** Paula Toro-Mujica

**Affiliations:** Instituto de Ciencias Agroalimentarias, Animales y Ambientales, Universidad de O’Higgins, Chile. Ruta I-90. Km1, San Fernando, Chile, 3070000

**Keywords:** Egg consumers, Consumer segmentation, Purchasing behavior, Nutritional knowledge

## Abstract

Egg consumer preferences are increasingly shaped by attributes beyond price, including perceived quality, nutritional value, and characteristics of the production system and production-related attributes. However, limited evidence is available on how these factors interact to define consumer profiles in Latin America. Therefore, this study examined egg purchasing and consumption behavior among surveyed consumers in central Chile (n = 412) by integrating purchasing habits, nutritional knowledge, perceptions of egg quality, and attitudes toward production systems. Given the marked predominance of young respondents (82.8% between 18 and 29 years old) and the digital channel through which the survey was administered, this study places particular emphasis on an emerging consumer segment characterized by greater exposure to information, higher familiarity with technology-mediated communication, and increasing awareness of nutritional and production-related attributes. Egg purchasing and consumption behavior was shaped by the interplay among sociodemographic characteristics, level of knowledge, and the importance assigned to both product- and production-related attributes. In addition to conventional quality cues, such as freshness and shell characteristics, respondents showed high familiarity with production systems and sustainability-related concepts, including animal welfare (86.9%), cage-free systems (82.8%), free-range systems (70.9%), and carbon footprint (64.3%), while fair trade practices, as an ethical/value-chain certification attribute (35.7%), and access to pasture, as a production-related certification attribute (33.0%), were among the most relevant certification-related aspect. The Multiple Correspondence Analysis (MCA) retained three dimensions explaining 78% of the total inertia. Cluster analysis identified four consumer profiles, representing 8.3%, 60.4%, 22.8%, and 8.5% of respondents, ranging from lower-involvement consumers with limited interest in differentiated attributes to more informed and demanding consumers who placed greater importance on nutritional, sensory, and ethical aspects. Discriminant analysis correctly classified 93.9% of respondents. The results suggest the presence of an emerging consumer profile associated with youth, digital information exposure, and greater knowledge of nutritional and production-system attributes. These findings highlight the heterogeneous nature of egg consumer behavior and suggest that knowledge and perceptions of egg quality and production systems are important drivers of market differentiation. The results may support the development of targeted communication, certification, and marketing strategies for differentiated egg products.

## Introduction

Egg consumption per capita has increased steadily worldwide over the past 60 years, rising from 3.5 kg per capita in 1960 to more than 10 kg in recent years (FAOSTAT, 2026). This trend has been attributed to several factors, including rising income levels that support greater consumption of animal protein, increased awareness of the nutritional value of eggs, and the growing popularity of high-protein, low-carbohydrate diets (Henchion et al., 2021; Réhault-Godbert et al., 2019). Although price remains one of the most important attributes influencing egg purchase decisions (Öğütcü and Elmas, 2020), consumer preferences are increasingly shaped by additional product, production-related, environmental, and value-chain attributes.

Beyond price, consumers may consider commercial and nutritional quality, production systems such as organic or free range production, production-related attributes such as animal welfare and food safety, environmental indicators such as carbon and water footprints, and ethical or value-chain attributes such as fair trade (De Marchi et al., 2024; Estévez-Moreno et al., 2025; Samman et al., 2009). This diversification of decision criteria reflects a shift toward more complex food choices, particularly among emergence of consumer segments who are more exposed to information and more concerned with health, ethical, and environmental aspects of food production. Within this context, increasing attention has been paid to emerging consumer segments that are younger, more digitally connected, and more exposed to food-related information (Grunert, 2005; Verbeke, 2005; La Ragione and Risitano, 2026). In this study, emerging consumers are understood as a segment shaped not only by changing preferences, but also by youth, greater exposure to digital information environments, and higher levels of knowledge may incorporate multiple dimensions into their purchasing decisions, including quality, health, ethical, and sustainability-related considerations (Pieniak et al., 2010; Asioli et al., 2017; Doan et al., 2025).

This behavior can be interpreted through frameworks such as the Theory of Planned Behavior, which proposes that beliefs influence attitudes and intentions, ultimately influencing behavior (Ajzen, 1991). This perspective has also been applied in recent studies on sustainable food consumption among younger generations (Doan et al., 2025).

Commercial egg quality is commonly evaluated through external attributes such as egg size, shell cleanliness, shell color, and shell hardness, as well as internal attributes such as albumen consistency (an indicator of freshness), yolk color, and taste (Hisasaga et al., 2020). These attributes often serve as direct quality cues during purchase and consumption. However, preferences for some of these attributes vary across countries and regions, particularly for egg size, shell color, and yolk color (Rondoni et al., 2020; Chen et al., 2023; Preisinger, 2018; Yang, 2021; Grashorn, 2016).

In contrast, nutritional quality is less directly observable, and information about it depends more strongly on labeling, prior knowledge, and consumer interpretation. Differentiated egg enriched with omega-3 fatty acids, vitamins, or antioxidants have attracted growing consumer interest because of their potential health benefits, particularly with respect to cardiovascular health and other physiological functions (Fraeye et al., 2012; Khan et al., 2021). Nevertheless, consumer understanding of nutritional quality is often partial and uneven, with consumers tending to focus on specific nutrients, such as protein, while overlooking other relevant components including fat quality and micronutrients (Grunert et al., 2010; Pieniak et al., 2010).

Production systems and production-related attributes also influence consumer evaluations of eggs. Eggs from free-range or organic systems are often associated with improved animal welfare, more natural feeding practices, and higher perceived quality (Al-Ajeeli et al., 2018; De Marchi et al., 2024; Estévez-Moreno et al., 2025; Sinclair et al., 2022; Sünder et al., 2022). At the same time, sustainability-related attributes, such as carbon footprint and environmental impact, may further influence purchasing decisions, especially among consumers who evaluate food products through ethical, environmental, and quality-related dimensions (Hartmann and Siegrist, 2017).

Despite the growing relevance of these attributes, consumer knowledge of sustainability, environmental impacts, and alternative production systems remains uneven, highlighting the need for public policies and communication strategies aimed at increasing awareness of environmentally friendly and animal welfare-oriented consumption behaviors (Peschel et al., 2016). For example, in southern Chile, 52% of respondents were unfamiliar with cage-free or free-range systems (Berkhoff et al., 2020). However, the increasing availability of food-related information through digital platforms may be changing consumer knowledge and expectations, particularly among younger and more digitally connected groups (Cruz-Cárdenas et al., 2025). Education, prior knowledge and experience also influence the importance assigned to product attributes, contributing to heterogeneous consumer profiles and to potential gaps between stated preferences and actual purchasing behavior (Areal et al., 2024; Asioli et al., 2017; Cao et al., 2021; Pieniak et al., 2010; Vermeir and Verbeke, 2006; Grunert, 2005).

In this context, the analysis of more informed and potentially emerging consumer segments becomes particularly relevant, as these groups may represent early adopters of differentiated products and provide insight into future changes in egg consumption patterns. Although research on egg consumer behavior has increased, several gaps remain. First, most studies have been conducted in Europe, North America, and Asia, with limited empirical evidence from Latin American contexts, where socioeconomic conditions, production systems, and market structures differ substantially. Second, previous research has predominantly examined consumer preferences, knowledge, or perceptions in isolation, without integrating these dimensions into a unified analytical framework. Third, few studies have explicitly linked consumer knowledge and perceptions of production systems, production-related attributes, and certification cues with multivariate segmentation approaches capable of identifying distinct and actionable consumer profiles. Fourth, limited attention has been paid to the characterization of emerging consumer segments, particularly in developing regions, where rapid changes in access to information and evolving food-related values may be reshaping consumption patterns. This perspective is particularly relevant in digitally connected young populations, in which the rapid circulation of information may accelerate the emergence of consumer segments with more complex decision-making criteria and greater sensitivity to nutritional, ethical, and production-related attributes. Therefore, the objective of this study was to develop an integrative analytical framework combining consumer knowledge, perceptions, purchasing behavior, and attitudes toward egg quality, production systems, and production-related attribute to identify consumer segments in central Chile, with particular emphasis on informed and potentially emerging consumer profiles.

## Materials and methods

### Data collection and respondents

A web-based questionnaire was developed using Google Forms and distributed through social media platforms, including Facebook, Twitter, and WhatsApp groups. Because the questionnaire was disseminated through digital platforms, the resulting sample was expected to include respondents with relatively high exposure to online information environments, a feature considered relevant for the identification of emerging consumer segments. Data collection began on October 15, 2023, and remained open until the minimum required sample size was reached.

Before large-scale distribution, a pilot survey was conducted to assess the functionality of the online questionnaire, as well as the clarity of the questions, the appropriateness of the response options, respondents’ understanding of the concepts used, and the time required to complete the survey.

The data collected were anonymous, as no personally identifiable information was requested. Participation was voluntary, and respondents could withdraw from the survey at any time. Before starting the questionnaire, participants were provided with a brief description of the study objectives and the purpose of the survey.

Only respondents who met the following criteria were included in the analysis: (i) being 18 years of age or older, (ii) residing in central Chile, and (iii) consuming eggs.

The minimum required sample size was calculated assuming an infinite population using the standard formula proposed by Cochran (1977) (Eq. (1)). This approach was considered appropriate because the population of central Chile is approximately 13.5 million inhabitants (INE, 2025).

(1) $$n=\frac{Z^{2}\cdot p\cdot q}{E^{2}}$$

Where:

• *n* is the required sample size.
• *Z* is the critical value of the standard normal distribution associated with the desired confidence level (95%).
• *p* is the expected probability of success. A conservative value (p = 0.5) was assumed to maximize the sample size
• *q* is the probability of failure (q = 1 - p)
• *E* is the margin of error

Particular attention was given to identifying consumer segments associated with differences in age, digital information exposure, and knowledge related to egg quality, production systems, and production-related or sustainability attributes.

### Questionnaire development

A variable operationalization framework was used to define the survey questions and to ensure consistency between the conceptual dimensions of egg consumer behavior and their measurable indicators. This framework enables the systematic organization and categorization of variables and their corresponding dimensions, thereby supporting questionnaire development, the definition of measurable indicators (Arias-González, 2021) and the subsequent multivariate analysis. In this study, six variables were considered, resulting in the identification of 14 dimensions and a total of 33 indicators (Table 1). These indicators were used to design the questionnaire, which consisted of 37 questions, including 32 closed-ended and 5 open-ended questions (Appendix 1). Table 1 presents the variable operationalization framework used in this study.

**Table 1:** Variable operationalization framework used for questionnaire design. The table presents the conceptual organization of the questionnaire, including the main variables, conceptual definitions, dimensions, and measurable indicators used to assess egg purchasing and consumption behavior.

*Variable*	*Conceptual definition*	*Dimension*	*Indicators*
Consumer profile	Sociodemographic characteristics of the consumer related to place of residence, education, and income level	Demographic profile	Age (1)* Gender (2) Municipality of residence (6) Number of household members (7)
		Socioeconomic profile	Occupation (3) Highest level of education completed (4) Relationship with rural life (5) Average monthly income (8)
Purchasing habits	Behavioral patterns and decision-making processes involved in egg purchase	Purchase-related decisions	Purchase responsibility (15) Purchase frequency (16) Purchase format (20) Place of purchase (21)
		Purchase criteria	Product origin (17) Size (18) Color (19) Product price (22) Relevant aspects in product choice (23)
Consumption habits	Behavioral patterns and preferences related to egg consumption.	Consumption-related decisions	Consumption occurrence (9) Consumption drivers (10) Consumption frequency (11) Consumption occasion (13) Quantity consumed (14)
		Consumption motives	Reasons for consumption (12)
Product knowledge	Information that the consumer possesses regarding the nutritional contribution of eggs.	Nutritional knowledge	Knowledge of nutritional contribution (24) Knowledge of myths about eggs (27) Perceived accuracy of information about eggs (28)
		Egg quality	Characteristics associated with egg quality (30)
Product valuation	Importance assigned by consumers to aspects related to the commercial and nutritional quality of the product.	Commercial quality attributes	Characteristics associated with yolk color (31) Characteristics associated with egg freshness (32)
		Nutritional contribution	Concern about nutritional contribution (25) Reasons for concern (26) Importance assigned to nutritional quality attributes (34)
		Willingness to pay	Accepted price increase for product certification (35)
Knowledge of production systems	Knowledge that respondents have regarding egg production systems and how these relate to product quality.	Knowledge	Knowledge of concepts related to production systems (29)
		Relevance	Relevant attributes of the production system (36)
		Certification	Importance of certifications (37)

* Numbers in parentheses correspond to the questionnaire item numbers included in Appendix 1.

### Data management and statistical analysis

The responses obtained from the questionnaire were exported to an Excel spreadsheet. Data processing included data cleaning, categorization of responses to open-ended questions, regrouping of infrequently selected response categories, and preparation of the dataset for subsequent analyses.

Descriptive statistics were generated using frequency tables for qualitative variables, particularly those related to respondents’ sociodemographic characteristics. Chi-square tests were performed to assess associations between sociodemographic characteristics and purchasing and consumption habits. A significance level of p < 0.05 was adopted for all statistical analyses.

#### Consumer segmentation

To examine the relationships among the variables underlying purchasing and consumption behavior among respondents who reported consuming eggs, and to identify consumer groups, a multivariate analysis was conducted.

The multivariate analysis comprised four stages: (1) variable selection, (2) Multiple Correspondence Analysis (MCA), (3) cluster analysis, and (4) discriminant analysis. The suitability of variables for inclusion in the MCA was evaluated using chi-square tests between pairs of variables. Variables showing significant associations with at least 50% of the total number of variables were retained (Vargas-Bello-Pérez et al., 2022).

The number of retained dimensions was determined based on eigenvalues greater than the mean eigenvalue (Greenacre, 2006), whereas Cronbach’s alpha was used to assess the internal consistency of each retained dimension (George and Mallery, 2003). The classification accuracy of the discriminant analysis was evaluated using cross-validation. Once consumer groups were defined, chi-square tests were performed to identify the key variables contributing to group differentiation. Statistical analyses were performed using R (R Core Team, 2023) within the RStudio environment (Posit team, 2023) and IBM SPSS Statistics (version 20.0; IBM Corp., 2011).

## Results

### Survey response and final sample size

Using the equation proposed by Cochran (1977) for estimating sample size in infinite populations, a Z value of 1.96 was adopted for a 95% confidence level. A conservative scenario was assumed with *p* = 0.5 and *q* = 0.5 to maximize the required sample size, in the absence of prior information on the proportion of consumers assigning importance to commercial quality attributes, nutritional quality, and egg origin. In addition, a margin of error (*E*) of 0.05 was considered. Under these assumptions, the minimum required sample size was estimated at 384 individuals.

This minimum number of respondents was reached within 15 days of launching the survey. However, to account for the potential presence of incomplete responses, the questionnaire remained open for an additional nine days, resulting in a total of 429 completed surveys.

Of these, 415 surveys were considered valid, corresponding to responses collected between October 15 and November 8, 2023. Three additional surveys were excluded from the multivariate analysis because fewer than 75% of the questions had been completed, resulting in a final analytical sample of 412 respondents, which exceeded the minimum requirement, thereby increasing the robustness of the analysis.

### Sample characteristics

A total of 110 variables were derived from the questionnaire responses, including eight sociodemographic variables. Regarding the sociodemographic profile, as shown in Table 2, 82.8% of respondents were between 18 and 29 years of age, while only 2.2% were older than 50 years. This age structure indicates that the study primarily captured a young consumer segment with a high likelihood of exposure to digital communication channels, which is relevant to the interpretation of the results in terms of emerging consumer profiles.

**Table 2:** Overall and group-specific sociodemographic characteristics of the identified consumer groups.

Characteristic	Group 1 8.3% (34)*	Group 2 60.4% (249)	Group 3 22.8% (94)	Group 4 8.5% (35)	Total 100% (412)
***Age***					
18–29 years	88.2% (30)	82.3% (205)	78.7% (74)	91.4% (32)	82.8% (341)
30–49 years	8.8% (3)	16.5% (41)	17.0% (16)	5.7% (2)	15.0% (62)
≥50 years	2.9% (1)	1.2% (3)	4.3% (4)	2.9% (1)	2.2% (9)
**Gender**					
Female	73.5% (25)	63.1% (157)	82.8% (77)	51.4% (18)	67.4% (277)
Male	26.5% (9)	36.9% (92)	17.2% (16)	48.6% (17)	32.6% (134)
***Household size***					
1	8.8% (3)	6.0% (15)	6.4% (6)	0.0% (0)	5.8% (24)
2–3	35.3% (12)	45.4% (113)	41.5% (39)	48.6% (17)	43.9% (181)
4–5	41.2% (14)	42.2% (105)	43.6% (41)	28.6% (10)	41.3% (170)
>5	14.7% (5)	6.4% (16)	8.5% (8)	22.9% (8)	9.0% (37)
***Municipality of residence***					
Mixed	29.4% (10)	30.9% (77)	29.8% (28)	34.3% (12)	30.8% (127)
Rural	11.8% (4)	8.4% (21)	10.6% (10)	11.4% (4)	9.5% (39)
Urban	58.8% (20)	60.6% (151)	59.6% (56)	54.3% (19)	59.7% (246)
***Education level***					
Primary or secondary education	38.2% (13)	32.5% (81)	24.5% (23)	42.9% (15)	32.0% (132)
Technical education or training	17.6% (6)	12.0% (30)	13.8% (13)	11.4% (4)	12.9% (53)
Bachelor’s degree	2.9% (1)	8.0% (20)	9.6% (9)	14.3% (5)	8.5% (35)
University degree	35.3% (12)	32.1% (80)	30.9% (29)	20.0% (7)	31.1% (128)
Postgraduate degree	5.9% (2)	15.3% (38)	21.3% (20)	11.4% (4)	15.5% (64)
***Employment status***					
Student (other fields)	35.3% (12)	36.5% (91)	28.7% (27)	54.3% (19)	36.2% (149)
Student (agronomy/veterinary medicine/animal production)	11.8% (4)	18.1% (45)	21.3% (20)	14.3% (5)	18.0% (74)
Employee or entrepreneur (other disciplines)	32.4% (11)	32.5% (81)	33.0% (31)	28.6% (10)	32.3% (133)
Employee or entrepreneur (agronomy/veterinary/animal production)	8.8% (3)	6.8% (17)	7.4% (7)	2.9% (1)	6.8% (28)
Other	11.8% (4)	6.0% (15)	9.6% (9)	0.0% (0)	6.8% (28)
***Experience with rural living***					
No	17.6% (6)	34.5% (86)	36.2% (34)	28.6% (10)	33.0% (136)
No, but frequent visits	23.5% (8)	15.7% (39)	18.1% (17)	22.9% (8)	17.5% (72)
Yes, lived previously	26.5% (9)	20.1% (50)	24.5% (23)	28.6% (10)	22.3% (92)
Yes, currently living in a rural area	32.4% (11)	29.7% (74)	21.3% (20)	20.0% (7)	27.2% (112)
***Monthly income***					
Less than USD 556	26.5% (9)	20.5% (51)	22.3% (21)	34.3% (12)	22.6% (93)
USD 556–1,111	55.9% (19)	36.1% (90)	29.8% (28)	31.4% (11)	35.9% (148)
USD 1,111–2,222	11.8% (4)	24.9% (62)	31.9% (30)	17.1% (6)	24.8% (102)
More than USD 2,222	5.9% (2)	18.5% (46)	16.0% (15)	17.1% (6)	16.7% (69)

*Values are presented as percentages and number of respondents in parentheses.

Overall, 67.4% of respondents were female, and 50.3% reported living in households with four or more members.

With respect to municipality of residence, 59.7% of respondents lived in urban municipalities, 30.8% in mixed municipalities, and only 9.5% in rural municipalities. However, when asked directly about their relationship with rural environments, 49.5% reported either currently living in or having previously lived in the countryside.

Approximately one-third of respondents (32.0%) completed primary or secondary education, a proportion similar to that of respondents holding a university degree (31.1%). In addition, 54.2% of respondents were students, with one-third of them enrolled in programs related to agronomy, veterinary sciences, or animal production. Meanwhile, 39.1% of respondents were either employers or employees, of whom 17.4% held positions related to agronomy, veterinary sciences, or animal production.

More than half of the respondents (58.5%) reported a monthly income below USD 1,111, whereas only 16.7% reported earning more than USD 2,222.

Appendix 2 summarizes the significant associations between sociodemographic variables and egg purchasing, consumption, and perception-related outcomes. This supplementary material provides additional context for interpreting the contribution of sociodemographic characteristics, while suggesting that consumer groups were not explained by these variables alone.

### Purchasing behavior

Regarding purchasing behavior, 40.8% of respondents reported being the person responsible for purchasing eggs. Eggs were most commonly purchased every two weeks (42.8%) or once per week (34.9%). Most consumers reported a preference for medium-sized eggs (53–62 g; 46.2%) and large eggs (63–72 g; 40.7%). The preferred purchasing format was trays of 30 eggs (64.5%) or 12 eggs (22.0%). Regarding shell color, white eggs were preferred by 46.2% of respondents, followed closely by brown eggs (43.1%). Eggs were purchased primarily from small-scale producers or farmers’ markets (32.7%), followed by minimarkets (16.2%) and supermarkets (15.0%).

**Fig. 1:**
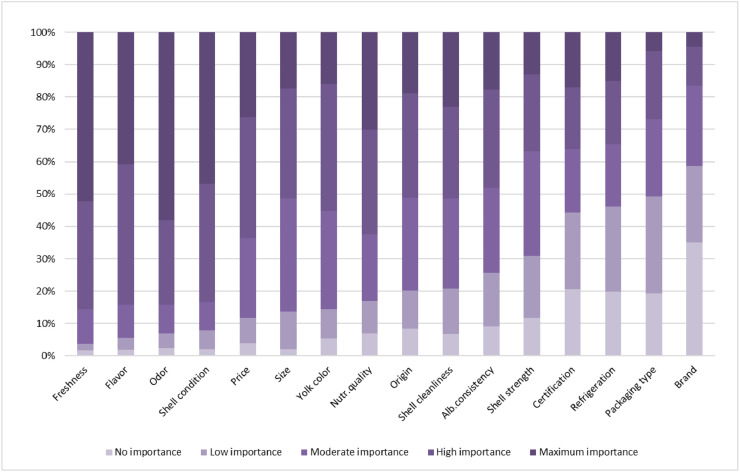
Importance assigned to egg purchase and perceived quality attributes by surveyed consumers. Values are expressed as percentages of respondents according to the response categories used in the questionnaire.

The five most valued attributes at the time of purchase were shell condition, price, size, shell cleanliness, and shell color (Fig. 1). In addition, the most important attributes associated with perceived quality were freshness, taste, odor, yolk color, and size (Fig. 1).

In terms of preferred production system, consumers showed a clear preference for eggs from backyard systems (42.5%) and free-range systems (25.4%), whereas industrially produced eggs were selected in only 19.0% of cases.

### Egg consumption and perceptions of quality and production systems and certification-related attributes

Regarding egg consumption, 18.0% of respondents reported consuming eggs daily. The most common consumption occasions were breakfast (80.3%) and dinner/evening meal (82.8%).

Consumers primarily associated commercial egg quality with attributes such as taste, yolk color, freshness, odor, and product origin. Yolk color was mainly associated with the hen’s diet, as well as with better appearance, improved taste, and higher perceived nutritional quality. Freshness, in turn, was primarily associated with expiration date, odor, and albumen consistency.

**Fig. 2:**
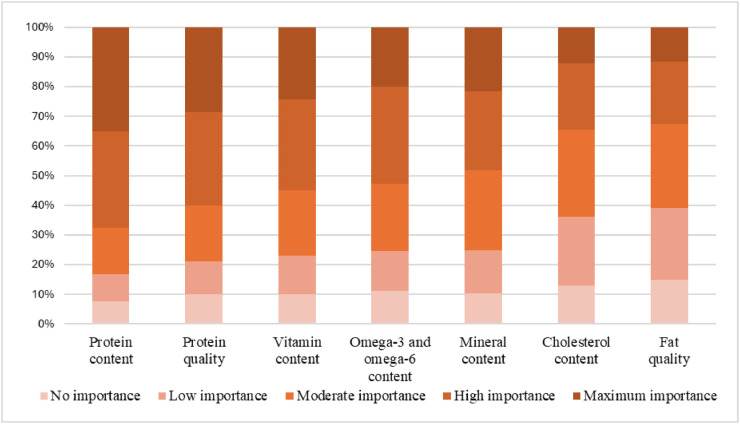
Importance assigned to nutritional quality attributes of eggs by surveyed consumers. Values are expressed as percentages of respondents according to the response categories used in the questionnaire.

The nutritional value of eggs was recognized by 77.0% of respondents and considered an important factor by 68.2%. Among the reasons for concern about nutritional value, 52.4% of consumers cited its relationship with health, whereas 27.8% highlighted protein content. In this context, respondents assigned the greatest importance to protein content and quality, whereas cholesterol content and fat quality were considered less important (Fig. 2).

In relation to production systems and production-related concepts, 86.9% of respondents reported being familiar with the concept of animal welfare, 82.8% with cage-free systems, 70.9% with free-range systems, and 64.3% with the concept of carbon footprint. Among production-related attributes, animal welfare (43.4%), family-farm production (26.5%), and access to pasture (23.1%) were the most valued. In addition, direct purchase from producers (33.0%) emerged as an important value-chain attribute.

Finally, with regard to certification, most respondents indicated a willingness to pay a premium of 5–10% for products certified as meeting specific production-related standards. The aspects considered most important for certification included cage-free conditions or access to outdoor areas as production-system certification criteria (38.8%), fair trade practices as an ethical/value-chain certification attribute (35.7%), and access to pasture as a production-related certification criterion (33.0%). Regarding the factors influencing purchasing decisions for certified eggs, respondents reported placing the greatest trust in the opinions of physicians or nutritionists (87.1%) and researchers or academics (86.7%), followed by animal welfare organizations (62.9%), foundations/NGOs (49.8%), and personal trainers (41.5%). In contrast, less influence was attributed to friends or family (39.6%), mass media (25.5%), industry stakeholders (23.5%), and social media influencers (9.5%).

### Consumer segmentation

The variable selection process resulted in the inclusion of 48 variables in the Multiple Correspondence Analysis (MCA). Three dimensions were retained, accounting for 78% of the total inertia. The overall Cronbach’s alpha was 0.94, indicating high internal consistency (Table 3).

**Table 3:** Summary of the Multiple Correspondence Analysis model.

Dimension	Cronbach’s alpha	Eigenvalue	Explained variance (inertia)
1	0.96	17.36	0.362
2	0.93	11.17	0.23
3	0.91	9.11	0.19
Total		37.64	0.78

**Fig. 3:**
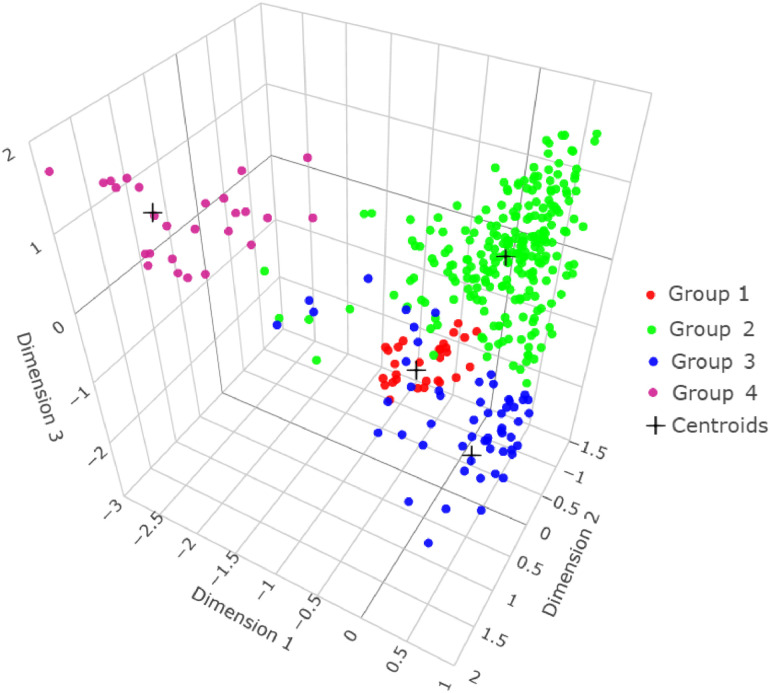
Three-dimensional representation of consumer clusters identified through Multiple Correspondence Analysis (MCA) and cluster analysis. The plot shows the distribution of respondents according to the first three MCA dimensions retained in the analysis. The four consumer profiles were identified using Ward’s clustering method with squared Euclidean distance.

The clustering method that yielded the best result was Ward’s method using squared Euclidean distance, identifying four groups that represented 8.3%, 60.4%, 22.8%, and 8.5% of respondents, respectively (Fig. 3). The discriminant analysis correctly classified 93.9% of the respondents. No misclassified individuals were observed in Groups 1 and 4.

Regarding sociodemographic variables, no significant differences were observed among groups in age, occupation, place of residence, or household size. However, significant differences were detected for gender (p = 0.03) and marginal differences for income level (p = 0.07) (Table 4). Gender differences were characterized by a higher proportion of female respondents in Groups 2 and 3. Similarly, income patterns suggested a tendency toward higher income levels in these same groups, although the differences did not reach the conventional threshold for statistical significance.

**Table 4:** Associations between consumer groups and selected sociodemographic characteristics, purchasing behavior, egg quality perceptions, nutritional attributes, production-related attributes, and value-chain attributes.

Category		Group 1	Group 2	Group 3	Group 4	p
Gender	Female	73.5	63.1*	82.8*	51.4*	0.03
	Male	26.5	36.9*	17.2*	48.6*	
Income	Less than USD 556	26.5	20.5	22.3	34.3	0.07
	USD 556–1,111	55.9*	36.1	29.8	31.4	
	USD 1,111–2,222	11.8	24.9	31.9	17.1	
	More than USD 2,222	5.9	18.5	15.9	17.1	
Person responsible for purchasing eggs	Yes	35.3	38.9	52.1*	28.6	0.06
	No	23.5	18.5	22.3	28.6	
	Sometimes	41.2	42.6*	25.5*	42.9	
*Importance assigned to...*						
Refrigeration	No importance	38.5*	12.8*	21.9	52.0*	<0.01
	Low importance	23.1	30.1	17.8	24.0	
	Moderate importance	26.9	22.2	13.7	4.0*	
	High importance	11.5	23.2*	17.8	4.0*	
	Maximum importance	0.0*	11.8*	28.8*	16.0	
Price	No importance	0.0	1.5*	5.5	24.0*	<0.01
	Low importance	11.5	6.9	6.9	12.0	
	Moderate importance	26.9	27.1	23.3	8.0*	
	High importance	30.8	43.8*	24.7*	28.0	
	Maximum importance	30.8	20.7*	39.7*	28.0	
Size	No importance	0.0	1.0	4.1	8.0*	<0.01
	Low importance	7.7	11.3	9.6	24.0*	
	Moderate importance	38.5	39.9*	21.9*	28.0	
	High importance	38.5	38.4*	24.7	20.0	
	Maximum importance	15.4	9.4*	39.7*	20.0	
Taste	No importance	0.0	0.4*	1.1	17.1*	<0.01
	Low importance	2.9	3.6	1.1	11.4*	
	Moderate importance	14.7	11.7	4.3*	11.4	
	High importance	47.1	54.2*	19.2*	28.6	
	Maximum importance	35.3	30.1*	74.5*	31.4	
Odor	No importance	0.0	1.2	1.1	17.1*	<0.01
	Low importance	11.8	3.2	4.3	8.6	
	Moderate importance	11.8	11.2*	2.1*	5.7	
	High importance	32.4	32.5*	11.7*	14.3	
	Maximum importance	44.1	51.8*	80.9*	54.3	
Yolk color	No importance	2.9	2.8*	5.3	25.7*	<0.01
	Low importance	23.5*	6.8	5.3	20.0*	
	Moderate importance	23.5	36.6*	19.2*	22.9	
	High importance	29.4	46.2*	30.9	22.9*	
	Maximum importance	20.6	7.6*	39.4*	8.6	
Shell color	No importance	2.9	10.4	14.9	25.7*	<0.01
	Low importance	29.4	18.9*	20.2	45.7*	
	Moderate importance	47.1	41.4*	25.5*	5.7*	
	High importance	20.6	24.9*	12.8*	17.1	
	Maximum importance	0.0	4.4*	26.6*	5.7	
Shell hardness	No importance	8.8	8.0*	8.5	48.6*	<0.01
	Low importance	35.3*	19.3	7.4*	34.3*	
	Moderate importance	38.2	38.6*	22.3*	8.6*	
	High importance	17.6	26.1	26.6	5.7*	
	Maximum importance	0.0	8.0	35.1	2.9	
Shell cleanliness	No importance	8.8	3.2*	7.4	42.9*	<0.01
	Low importance	17.6	15.7	6.4*	14.3	
	Moderate importance	38.2	33.3*	16.0*	20.0	
	High importance	23.5	34.9*	21.3	8.6*	
	Maximum importance	11.8	12.9*	48.9*	14.3	
Product origin	No importance	8.8	4.8*	6.4	40.0*	<0.01
	Low importance	26.5*	8.4*	11.7	20.0	
	Moderate importance	50.0*	32.1	16.0*	17.1	
	High importance	8.8*	41.8*	22.3*	14.3*	
	Maximum importance	5.9	12.9	43.6	8.6	
Access to pasture	No importance	2.9	1.6*	2.1	54.3*	<0.01
	Low importance	41.2*	10.4*	7.4	22.9	
	Moderate importance	50.0*	33.7*	12.8*	5.7*	
	High importance	5.9*	40.2*	13.8*	17.1	
	Maximum importance	0.0*	14.1*	63.8*	0.0*	
Animal welfare	No importance	0.0	0.4*	2.1	28.6*	<0.01
	Low importance	26.5*	4.0*	2.1*	22.9*	
	Moderate importance	26.5	19.7	6.4*	14.3	
	High importance	32.4	39.8*	5.3*	20.0	
	Maximum importance	14.7*	36.1*	84.0*	14.3*	
Family-farm production	No importance	0.0	0.8*	2.1	40.0*	<0.01
	Low importance	32.4*	5.6*	2.1*	22.9*	
	Moderate importance	32.4	29.7*	11.7*	17.1	
	High importance	29.4	45.0*	20.2*	20.0*	
	Maximum importance	5.9*	18.9*	63.8*	0.0*	
Certification	No importance	34.6	16.8*	12.3	60.0*	<0.01
	Low importance	30.8	25.6	19.2	16.0	
	Moderate importance	15.4	23.7*	13.7	8.0	
	High importance	3.9*	22.2	19.2	8.0	
	Maximum importance	15.4	11.8*	35.6*	8.0	
Nutritional quality	No importance	5.9	2.4*	3.2	51.4*	<0.01
	Low importance	41.2*	7.2*	1.1*	22.9*	
	Moderate importance	47.1*	23.7	4.3*	17.1	
	High importance	5.9*	47.8*	11.7*	2.9*	
	Maximum importance	0.0*	18.9*	79.8*	5.7*	
Freshness	No importance	5.9	0.4*	0.0	11.4*	<0.01
	Low importance	0.0	1.2	0.0	14.3*	
	Moderate importance	17.6	10.8	1.1*	28.6*	
	High importance	44.1	40.6*	13.8*	25.7	
	Maximum importance	32.4*	47.0*	85.1*	20.0	
Albumen consistency	No importance	8.8	6.8*	4.3*	40.0	<0.01
	Low importance	26.5	16.9	5.3*	34.3*	
	Moderate importance	29.4	31.3*	16.0*	14.3	
	High importance	23.5	35.7*	27.7	5.7*	
	Maximum importance	11.8	9.2*	46.8*	5.7	
*Nutritional contribution*						
Concern about it	Yes	23.5*	75.9*	84.0*	14.3*	<0.01
Knowledge of it	Yes	55.9*	81.9*	90.4*	34.3*	<0.01
*Knowledge of production-system concepts…*						
Free-range systems	Yes	52.9*	75.1*	73.4	51.4*	<0.01
Carbon footprint	Yes	38.2*	67.9	64.9	62.9	<0.01
*Associates yolk color with...*						
Hen feeding	No	55.9	40.2*	50.0	82.9*	<0.01
	Yes	44.1	59.8*	50.0	17.1*	
*Associates freshness cue with...*						
Floating test	No	67.6*	40.6	36.2	65.7*	<0.01
	Yes	32.4*	59.4	63.8	34.3*	

Values are expressed as percentages. An asterisk (*) denotes values that differ significantly from the overall sample distribution based on adjusted residuals (p < 0.05).

With respect to purchasing and consumption behavior, differences among groups were identified in the reasons for egg consumption, daily consumption occasions, and the importance assigned to several product attributes, including (i) refrigeration practices, (ii) purchase price, (iii) shell color, (iv) shell cleanliness, (v) packaging, (vi) shell condition, (vii) egg size, (viii) brand, and (ix) certification. In particular, Group 3 included a higher proportion of individuals directly involved in egg purchasing, suggesting a more active role in decision-making processes.

Significant differences (p < 0.01) were also observed in the importance assigned to both intrinsic and extrinsic quality attributes. Group 3 consistently placed greater importance on sensory attributes such as taste, odor, yolk color, and freshness, as well as on physical indicators including shell cleanliness, shell hardness, and albumen consistency. In contrast, Group 4 assigned lower importance to most of these attributes, indicating a less quality-oriented consumption pattern, whereas Group 2 showed an intermediate profile with a greater emphasis on price-related considerations.

Differences were also found in respondents’ concern about nutritional quality, as well as in their knowledge of production systems such as free-range systems and sustainability-related concepts such as carbon footprint. Variation was further observed in perceptions of quality attributes, including the presence of spots in the yolk or albumen and egg size. In addition, groups differed in their knowledge and perceptions related to sustainable production and animal welfare, factors that may ultimately influence both consumption levels and the types of eggs consumed (Table 4).

These differences also extended to production system attributes. Group 3 assigned the highest importance to animal welfare, access to pasture, and family-farm production, whereas Group 4 consistently rated these aspects as less relevant. Similarly, certification and nutritional quality were more highly valued by Group 3, while Groups 1 and 4 showed comparatively lower levels of concern.

Finally, significant variation was observed in knowledge and perceptions related to egg quality and production systems. Group 3 demonstrated higher levels of awareness regarding nutritional contribution and production concepts such as free-range systems and carbon footprint, whereas Group 4 exhibited lower levels of knowledge and concern across these dimensions. Overall, these patterns indicate the presence of distinct consumer profiles characterized by differences in quality perception, knowledge, and production system preferences, which are likely to influence both purchasing decisions and consumption behavior (Table 4).

The significant differences identified among groups allowed the characterization of four consumer profiles.

**Group 1. Traditional profile with low orientation toward differentiated attributes.**

This group was characterized by medium income levels, limited knowledge of the nutritional contribution of eggs, and lower importance assigned to this attribute, as well as to egg certification. Accordingly, respondents in this group assigned low importance to specific nutritional components, including fat quality, unsaturated fatty acids, cholesterol content, protein content and quality, and vitamins and minerals. In addition, this group showed limited awareness of concepts such as carbon footprint and free-range systems and placed less importance on product origin and production-related, ethical, and value-chain attributes, including animal welfare, access to pasture, family-farm production, and fair trade practices. They also assigned lower importance to freshness-related indicators, such as albumen consistency, despite reporting that freshness was a relevant attribute.

**Group 2. Intermediate profile with moderate knowledge and valuation of attributes.**

This group exhibited a higher proportion of male respondents and individuals who occasionally took responsibility for purchasing eggs. Attributes such as refrigeration, freshness, egg size, price, and product origin were considered highly important. In addition, respondents in this group demonstrated awareness of and concern about the nutritional contribution of eggs, including protein content and quality, fat quality, cholesterol, vitamin and mineral content. Certification was considered moderately important. More than 70% of individuals in this group indicated a willingness to pay a premium of 5–20% for eggs with quality certifications.

**Group 3. Demanding, informed, and quality-oriented profile.**

This group was predominantly composed of women with above-average income levels, a pattern that may be associated with the relatively lower importance assigned to price. Respondents in this group showed a strong concern for both egg quality and production-related certifications, although attributes such as egg size and shell color were considered less relevant. They demonstrated a high level of knowledge regarding the nutritional contribution of eggs and assigned maximum importance to key quality attributes such as taste, odor, yolk color, albumen consistency, shell cleanliness, product origin, and animal welfare. Furthermore, this group placed the highest importance on specific nutritional components, including fat quality, cholesterol, omega-3 and omega-6 fatty acids, protein content and quality, and vitamins and minerals. They also reported a greater reliance on the opinions of researchers and academics when considering certified products in their purchasing decisions.

**Group 4. Low-involvement profile toward differentiated attributes.**

Although women accounted for 51.4% of this group, it was characterized by lower levels of concern about and knowledge of nutritional aspects. It showed the lowest proportion of individuals aware of the nutritional contribution of eggs and assigned low importance to attributes such as access to pasture. This group concentrated the highest proportions of responses in the “no importance” or “low importance” categories across several product-related variables including refrigeration, certification, taste, yolk color, product origin, nutritional quality, shell hardness, albumen consistency, shell cleanliness. Low importance was also assigned to production-related attributes, such as access to pasture, animal welfare, family-farm production, as well as to commercialization attributes, such as direct purchase from producers and fewer intermediaries. Similarly, it assigned low importance to specific nutritional components such as fat, cholesterol, omega-3 and omega-6 fatty acids, protein, vitamins, and minerals. Moreover, this group showed the lowest level of concern about nutrient content and the highest proportion of respondents unwilling to pay a premium price. Overall, this profile is consistent with a lower level of engagement with differentiated attributes and a lower responsiveness to technical information or value-added product characteristics.

## Discussion

The results indicate that egg purchasing and consumption behavior among surveyed consumers was shaped by the interaction sociodemographic characteristics, nutritional knowledge, perceptions product quality, and attitudes toward production systems, and production-related attributes. This multidimensional decision-making process is consistent with previous studies showing that food choices influenced not only by economic factors but also by cognitive and attitudinal components (Grunert, 2005; Verbeke, 2005; Rondoni et al., 2020; Öğütcü and Elmas, 2020). From the perspective of the Theory of Planned Behavior, these findings suggest that beliefs and perceptions regarding egg quality, nutrition, and production practices may contribute to shaping consumer attitudes and purchase-related decisions (Vermeir and Verbeke, 2006; Ajzen, 1991).

The high proportion of young respondents may partly explain the relatively high awareness observed for concepts related to sustainability, animal welfare, alternative production systems and ethical production practices. Rather than representing only an age effect, this pattern may reflect the role of digital information exposure in shaping more informed food-related attitudes. In this sense, the surveyed population can be interpreted as capturing an emerging consumer segment characterized by greater access to information and a stronger tendency to integrate nutritional, ethical, environmental, and production-related criteria into purchasing decisions (Hartmann and Siegrist, 2017). This interpretation is consistent with evidence showing that information exposure influences consumer perceptions and food choices (Peschel et al., 2016), although knowledge remains unevenly distributed across consumer groups.

Regarding purchasing behavior, the results confirm that traditional quality cues remain highly relevant. Attributes such as shell condition, cleanliness, egg size, and freshness function as immediate indicators of quality and facilitate consumer evaluation at the point of purchase, as previously reported for egg and food choice behavior (Rondoni et al., 2020). However, these conventional attributes coexist with a high valuation of production-related and extrinsic attributes, including animal welfare, product origin, certification and preference for eggs from backyard or free-range systems. This pattern supports the idea that some consumers associate alternative production systems with naturalness, safety, higher product quality, and improved animal welfare (Doyon et al., 2023; Sinclair et al., 2022). Similar trends have been reported in Europe and North America, where consumers increasingly associate production practices with ethical and quality-related values (Doyon et al., 2023; De Marchi et al., 2024).

Nevertheless, the relevance assigned to differentiated attributes should be interpreted with caution, because stated preferences do not necessarily translate into actual purchasing behavior, particularly when price remains a limiting factor. This attitude–behavior gap has been widely documented for ethically differentiated food products (Vermeir and Verbeke, 2006). Therefore, although the results suggest a transition from a predominantly price-oriented approach toward a broader valuation of quality and production-related attributes, this transition appears to be heterogeneous and dependent on consumer knowledge, income, and level of involvement.

Nutritional knowledge also emerged as a key determinant of consumer behavior. A high proportion of respondents reported being aware of the nutritional value of eggs and expressed concern about this aspect, particularly regarding protein content. This finding is consistent with the literature, which positions eggs as a high-quality protein source highly valued by consumers (Öğütcü and Elmas, 2020). However, the lower importance assigned to fat quality and cholesterol suggests that nutritional understanding may remain partial or focused on simplified nutritional cues (Grunert et al., 2010). This finding is consistent with previous studies showing that consumers often rely on selected nutrients rather than comprehensive nutritional evaluations (Grunert et al., 2010; Pieniak et al., 2010). Therefore, communication strategies should address not only the protein value of eggs, but also broader aspects of nutritional quality.

The identification of four consumer profiles highlights the heterogeneity of egg consumer behavior and confirms the value of integrating purchasing habits, nutritional knowledge, quality perception, and attitudes toward production systems into a multivariate segmentation framework.

**Fig. 4:**
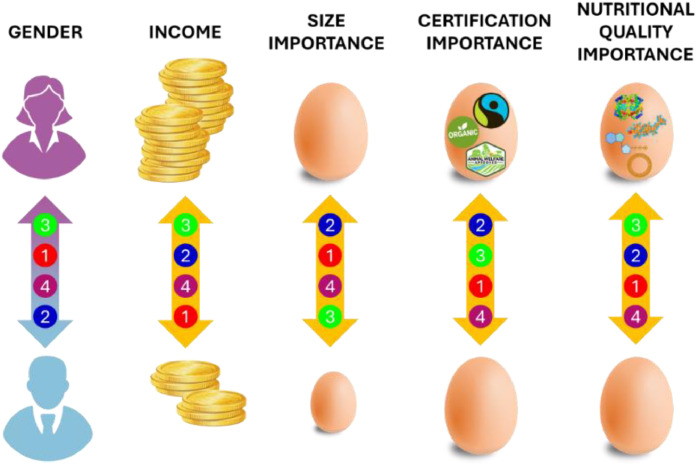
Conceptual representation of the main factors influencing egg purchasing decision-making among surveyed consumers.

These findings contribute to the literature by demonstrating that segmentation in egg markets is driven not only by observable preferences, but also by the interaction between nutritional knowledge, perceived product quality, attitudes toward production systems and production-related attributes, underscoring the importance of integrating cognitive and perceptual dimensions into the analysis of food choice behavior (Grunert, 2005; Asioli et al., 2017) (Fig. 4).

The “demanding and informed” profile (Group 3) showed the strongest valuation of sensory, nutritional, ethical, and production-related aspects suggesting that this group may represent the clearest expression of the emerging consumer segment identified in this study: a younger, more informed, and digitally exposed segment that assigns greater value to nutritional, sensory, and production-related attributes. This profile, composed predominantly of women and individuals with relatively higher income levels (Table 2, Table 4), is consistent with consumer segments described in the literature as “ethical” or “conscious,” who are generally more willing to pay premium prices for differentiated products (Vermeir and Verbeke, 2006). In contrast, the “traditional” and “low-involvement” profiles (Groups 1 and 4) showed lower levels of knowledge and weaker valuation of differentiated attributes, indicating that their purchasing decisions may be more strongly driven by habit, price or lower engagement with value-added characteristics. This pattern has been widely reported in international studies, where consumer knowledge and prior experience significantly influence the importance assigned to product attributes (Pieniak et al., 2010).

The intermediate profile (Group 2) is particularly relevant from a practical perspective, because its moderate knowledge and willingness to pay suggest potential responsiveness to information-based interventions (Öğütcü and Elmas, 2020; Cao et al., 2021). This profile may represent a transitional segment within this process, suggesting that emerging consumer behavior is not dichotomous, but develops gradually as access to information and familiarity with differentiated attributes increase.

The results are consistent with a shift from a predominantly price-oriented consumption pattern toward a broader valuation of quality-related attributes. However, this transition is not homogeneous across consumer segments, highlighting the need for differentiated strategies from both producers and policymakers Efforts aimed at improving consumer education, strengthening certification systems, and increasing transparency in production practices may therefore support the development of differentiated egg markets. The findings should be interpreted primarily as reflecting a young, digitally connected surveyed population rather than the entire population of egg consumers in central Chile. A broader understanding of the full population of egg consumers requires more representative sampling designs in which differences in access to technology are not a source of bias, in addition to combining stated preferences with observed behavior, particularly to better understand the persistence of the attitude–behavior gap in food consumption (Vermeir and Verbeke, 2006).

## Conclusion

The results of this study suggest that egg purchasing and consumption behavior among surveyed consumers was shaped by the interplay among sociodemographic characteristics, nutritional knowledge, perceived egg quality, and attitudes toward production systems and production-related attributes. Although price remained relevant, respondents showed a clear valuation of attributes associated with commercial and nutritional quality, animal welfare, product origin, and certification.

The identification of four consumer profiles highlights the heterogeneity among surveyed egg consumers, ranging from low-involvement groups with limited interest in differentiated attributes to informed and demanding consumers who placed greater importance on nutritional, sensory, and ethical aspects. The more quality-oriented profile suggests the presence of an emerging consumer segment associated with youth, digital information exposure, and greater awareness of nutritional and production-related attributes.

Overall, the findings point to an ongoing shift from price-driven consumption patterns toward a more holistic valuation of egg quality. However, this transition was not uniform across consumer groups, highlighting the need for targeted communication, certification, and marketing strategies for differentiated egg products. The design of these strategies could be supported by the findings of this study.

## Author contributions

**Paula Toro-Mujica:** Conceptualization, Methodology, Investigation, Data curation, Formal analysis, Visualization, Writing - original draft, Writing - review & editing, Project administration, Funding acquisition.

The author confirms that she is the sole author of this manuscript and takes responsibility for all aspects of the work.

## Disclosures

The author listed below certifies that she has no affiliations with or involvement in any organization or entity with any financial interest (including honoraria, educational grants, participation in speakers’ bureaus, membership, employment, consultancies, stock ownership or other equity interests, and expert testimony or patent-licensing arrangements) or non-financial interest (including personal or professional relationships, affiliations, knowledge, or beliefs) in the subject matter or materials discussed in this manuscript.

## Appendix Supplementary materials

Image, application 1
