# Supplementary material for: Consumer segmentation and drivers of egg purchasing behavior among emerging consumers in central Chile: The role of nutritional knowledge, production systems, and quality perception
Source: Poult Sci. 2026 May 22;105(9):107161. doi: 10.1016/j.psj.2026.107161 (PMC13265865; doi:10.1016/j.psj.2026.107161)
Supplement: Supplementary file 1 [file mmc1.docx]

**Appendix 1. Questionnaire used to assess egg purchasing behavior, consumption habits, nutritional knowledge, and perceptions of egg quality and production systems**

(The questionnaire included automated skip logic programmed within the online survey platform, so respondents were automatically routed to the appropriate subsequent question according to their previous response)

**1. Age ***

*Response type: Open-ended*

**2. Gender ***

*Response type: Single choice*

- Male
- Female
- Prefer not to say

**3. Occupation ***

*Response type: Single choice*

- Student (agronomy/veterinary/animal production/zootechnics)
- Student (other disciplines)
- Business owner or employee (agronomy/veterinary/animal production/zootechnics)
- Business owner or employee (other disciplines)
- Retired (other disciplines)
- Unemployed
- Homemaker

**4. What is the highest level of education you have completed? ***

*Response type: Single choice*

- Primary education
- Secondary education
- Technical education/training
- Bachelor’s degree
- University degree
- Specialization, diploma, master’s degree
- Doctorate
- Other

**5. Do you live or have you lived in the countryside? ***

*Response type: Single choice*

- Yes, I currently live there
- Yes, I have lived there
- No, but I travel there frequently
- No

**6. Municipality of residence ***

*Response type: Open-ended*

**7. Number of people living in the household ***

*Response type: Single choice*

- 1
- 2
- 3
- 4
- 5
- More than 5

**8. Average monthly income ***

*Response type: Single choice*

- Less than USD 556
- USD 556–1,111
- USD 1,111–2,222
- More than USD 2,222

**9. Do you consume eggs? ***

*Response type: Single choice*

- Yes (skip to question 11)
- No (skip to question 10)

**10. Why do you NOT consume eggs? ***

*Select up to 3 options.*

*Response type: Multiple selection (maximum 3)*

- It is not part of my diet
- I do not like them
- Because of health problems
- Because of how animals are treated
- Concern for the environment
- Other

**11. How often do you consume eggs? ***

*Response type: Single choice*

- Daily
- 1-3 times per week
- Once a week
- Once every two weeks
- Once a month
- A few times a year
- Never

**12. Why do you consume eggs? ***

*Response type: Multiple selection (maximum 3)*

- Because I like the taste
- Because they are healthy
- Because they provide nutrients
- Out of habit
- Because they are easy to prepare
- Because they are a low-cost source of protein
- Because many recipes require them
- To vary my diet

**13. At what time(s) do you consume eggs?**

*Response type: Multiple selection (maximum 3)*

- Breakfast
- Snack
- Lunch
- Dinner/evening meal
- During outings or excursions

**14. How many eggs do you consume on each occasion? ***

*Response type: Single choice*

- 1
- 2
- 3
- 4

**15. Do you buy eggs for your household? ***

*Response type: Single choice*

- Yes (skip to question 16)
- Sometimes (skip to question 16)
- No (skip to question 24)

**16. How often do you buy eggs? ***

*Response type: Single choice*

- Every day
- Every other day
- Once a week
- Every 2 weeks
- Once a month

**17. What type of eggs do you prefer? ***

*Response type: Single choice*

- Industrial eggs
- Backyard eggs
- Eggs from small/medium-sized producers
- Free-range eggs
- Pasture-raised hen eggs

**18. What egg size do you buy? ***

*Response type: Single choice*

- Small
- Medium
- Large
- Extra large
- Jumbo/Special

**19. What egg color do you prefer? ***

*Response type: Single choice*

- White
- Brown
- Blue
- Green

**20. In what format do you buy eggs? ***

*Response type: Single choice*

- By unit
- Tray of 6 units
- Tray of 12 units
- Tray of 30 units
- Box (180 eggs)

**21. Where do you prefer to buy eggs? ***

*Response type: Single choice*

- Large supermarkets
- Minimarket or local supermarket
- Small producers/farmers’ market
- Open-air market
- Neighborhood store
- Poultry stores
- Distributor

**22. How much do you pay per unit? ***

*Response type: Open-ended*

**23. At the time of purchase, how much importance do you assign to the following factors? ***

| Factor | No importance | Low importance | Moderate importance | High importance | Maximum importance |
| --- | --- | --- | --- | --- | --- |
| Shell condition | ☐ | ☐ | ☐ | ☐ | ☐ |
| Price | ☐ | ☐ | ☐ | ☐ | ☐ |
| Size | ☐ | ☐ | ☐ | ☐ | ☐ |
| Shell cleanliness | ☐ | ☐ | ☐ | ☐ | ☐ |
| Shell color | ☐ | ☐ | ☐ | ☐ | ☐ |
| Certification | ☐ | ☐ | ☐ | ☐ | ☐ |
| Refrigeration | ☐ | ☐ | ☐ | ☐ | ☐ |
| Packaging type | ☐ | ☐ | ☐ | ☐ | ☐ |
| Brand | ☐ | ☐ | ☐ | ☐ | ☐ |
| Freshness | ☐ | ☐ | ☐ | ☐ | ☐ |
| Taste | ☐ | ☐ | ☐ | ☐ | ☐ |
| Yolk color | ☐ | ☐ | ☐ | ☐ | ☐ |
| Nutritional quality | ☐ | ☐ | ☐ | ☐ | ☐ |
| Product origin | ☐ | ☐ | ☐ | ☐ | ☐ |
| Shell hardness | ☐ | ☐ | ☐ | ☐ | ☐ |

**24. Do you know the nutritional contribution of eggs? ***

Response type: Single choice

- Yes
- No

**25. Are you concerned about the nutritional contribution that eggs provide? ***

*Response type: Single choice*

- Yes
- No

**26. Regarding the previous question, why?**

*Response type: Open-ended*

**27. Do you know any myths about eggs? Which one(s)?**

*Response type: Open-ended*

**28. Regarding the following myths/statements about eggs, indicate whether you consider them true or false.**

| **Statement** | **True** | **False** |
| --- | --- | --- |
| Eating eggs increases cardiovascular disease. | ☐ | ☐ |
| It is advisable for athletes and physically active people to consume eggs. | ☐ | ☐ |
| Eating raw eggs is dangerous. | ☐ | ☐ |
| Eggs should not be washed before storing them. | ☐ | ☐ |
| Eggs should be kept refrigerated. | ☐ | ☐ |
| Eggs from outdoor systems (free-range or organic) are more expensive. | ☐ | ☐ |
| Eggs from backyard hens are better than those from commercial farms. | ☐ | ☐ |

**29. Do you know or have you heard about the following concepts associated with egg production?**

| **Production-system concepts** | **Yes** | **No** |
| --- | --- | --- |
| Cage-free systems | ☐ | ☐ |
| Free-range systems (pasture access) | ☐ | ☐ |
| Carbon footprint | ☐ | ☐ |
| Animal welfare | ☐ | ☐ |

**30. Which of the following characteristics allow you to perceive whether an egg is of high quality? ***

*Response type: Multiple selection (maximum 3)*

- Taste
- Yolk color
- Shell color
- Odor
- Product origin
- Nutritional quality
- Shell hardness
- Albumen consistency
- Shell cleanliness
- Size
- Freshness
- Spots on the yolk or albumen

**31. What do you associate yolk color with?**

*Response type: Multiple selection (maximum 3)*

- Better nutritional quality
- Better taste
- Better appearance
- Production system
- Hen feeding
- Fresher egg

**32. Which characteristic(s) allow you to know that an egg is fresh? ***

*Response type: Multiple selection (maximum 3)*

- Albumen consistency
- Floating test
- Odor
- Shell cleanliness
- Expiration date
- Yolk color

**33. Which attributes of the production system are relevant to you? ***

| **Attribute** | **No importance** | **Low importance** | **Moderate importance** | **High importance** | **Maximum importance** |
| --- | --- | --- | --- | --- | --- |
| Access to pasture | ☐ | ☐ | ☐ | ☐ | ☐ |
| Animal welfare | ☐ | ☐ | ☐ | ☐ | ☐ |
| Family farm production | ☐ | ☐ | ☐ | ☐ | ☐ |
| Direct purchase from producer | ☐ | ☐ | ☐ | ☐ | ☐ |
| Fewer intermediaries | ☐ | ☐ | ☐ | ☐ | ☐ |

**34. Indicate the degree of importance you assign to the following aspects related to the nutritional quality of eggs. ***

| **Aspect** | **No importance** | **Low importance** | **Moderate importance** | **High importance** | **Maximum importance** |
| --- | --- | --- | --- | --- | --- |
| Protein content | ☐ | ☐ | ☐ | ☐ | ☐ |
| Protein quality | ☐ | ☐ | ☐ | ☐ | ☐ |
| Vitamin content | ☐ | ☐ | ☐ | ☐ | ☐ |
| Omega-3 and omega-6 content | ☐ | ☐ | ☐ | ☐ | ☐ |
| Mineral content | ☐ | ☐ | ☐ | ☐ | ☐ |
| Cholesterol content | ☐ | ☐ | ☐ | ☐ | ☐ |
| Fat quality | ☐ | ☐ | ☐ | ☐ | ☐ |

**35. What percentage increase in egg price would you be willing to pay for the certification of aspects associated with egg quality? ***

*Response type: Single choice*

- None
- Between 5-10%
- Between 10-20%
- Between 20-30%
- Between 30-40%
- Up to 50%
- Whatever is necessary

**36. Which aspects associated with quality assurance/certification do you consider most important? ***

*Response type: Multiple selection (maximum 3)*

- Fair trade
- Access to pasture
- Cage-free systems
- Access to outdoor areas
- Omega-3 or omega-6 fatty acid content (unsaturated fatty acids)
- Vitamin content
- Mineral content
- Amino acid content
- Low carbon footprint
- Product origin
- Organic production
- Sustainable/environmentally friendly production
- Drug-free production (hormones and antibiotics)

**37. Which opinions would you consider when deciding whether or not to buy eggs with quality certifications/assurance? ***

| **Source of opinion** | **Yes** | **No** |
| --- | --- | --- |
| Doctors or nutritionists | ☐ | ☐ |
| Researchers or academics | ☐ | ☐ |
| Friends or family | ☐ | ☐ |
| Foundations or NGOs | ☐ | ☐ |
| Media outlets | ☐ | ☐ |
| Animal welfare organizations | ☐ | ☐ |
| Companies or trade associations | ☐ | ☐ |
| Government officials or public institutions | ☐ | ☐ |
| Fitness trainers or personal trainers | ☐ | ☐ |
| Celebrities/influencers | ☐ | ☐ |
| None | ☐ | ☐ |

**Appendix 2.** Summary of significant associations between sociodemographic variables and egg purchasing, consumption, and perception-related outcomes

| **Sociodemographic variable** | **Number of significant associations (p < 0.05)** | **Main significant outcomes** |
| --- | --- | --- |
| Age group | 21 | Person responsible for purchasing eggs, consumes eggs at dinner/evening meal, preferred egg size, would consider opinions of personal trainers, associates yolk color with a fresher egg, consumes eggs as snack, importance of fair trade certification, egg consumption frequency, associates yolk color with better appearance, concern about nutritional contribution, uses yolk color as freshness cue, uses spots in yolk/albumen as quality cue, importance of omega-3 and omega-6 content, consumes eggs at breakfast, knows the nutritional contribution of eggs, importance of brand, would consider the opinions of foundations/NGOs, would consider the opinions of doctors/nutritionists, preferred shell color, importance of access to pasture, associates yolk color with hen feeding. |
| Gender | 12 | Consumes eggs at lunch, consumes eggs at dinner/evening meal, consumes eggs during outings or excursions, number of eggs consumed per occasion, knows carbon footprint concept, importance of shell color, importance of albumen consistency, importance of freshness, associates yolk color with better nutritional quality, willingness to pay price increase for certification, would consider the opinions of foundations/NGOs, would consider the opinions of animal welfare organizations. |
| Education level | 14 | Person responsible for purchasing eggs, associates yolk color with a fresher egg, preferred egg size, would consider the opinions of researchers or academics, concern about nutritional contribution, importance of cage-free/outdoor-access certification, knows carbon footprint concept, consumes eggs at dinner/evening meal, uses yolk color as freshness cue, importance of fair trade certification, importance of shell color, uses spots in yolk/albumen as quality cue, importance of fat quality, egg purchase frequency. |
| Occupation | 27 | Person responsible for purchasing eggs, consumes eggs at dinner/evening meal, knows the nutritional contribution of eggs, importance of fair trade certification, importance of shell cleanliness, egg consumption frequency, associates yolk color with a fresher egg, uses the floating test as a freshness cue, knows carbon footprint concept, willingness to pay price increase for certification, preferred shell color, knows the concept of cage-free systems, uses spots in yolk/albumen as quality cue, associates yolk color with hen feeding, would consider the opinions of personal trainers, importance of fewer intermediaries, price paid per unit, would consider the opinions of friends or family, importance of direct purchase from producer, importance of animal welfare, preferred egg size, importance of shell hardness, egg purchase frequency, importance of brand, importance of access to pasture, uses shell cleanliness as quality cue. |
| Income | 27 | Egg consumption frequency, consumes eggs as snack, person responsible for purchasing eggs, egg purchase frequency, preferred egg size, preferred shell color, preferred purchase format, price paid per unit (category), importance of price, knows the nutritional contribution of eggs, concern about nutritional contribution, knows the concept of cage-free systems, knows carbon footprint concept, number of production concepts known, importance of nutritional quality, associates yolk color with a fresher egg, associates yolk color with production system, uses the floating test as a freshness cue, uses shell cleanliness as freshness cue, importance of access to pasture, importance of fewer intermediaries, importance of fat quality, importance of omega-3 and omega-6 content, importance of fair trade certification, importance of cage-free/outdoor-access certification, would consider the opinions of animal welfare organizations, would consider the opinions of government officials or public institutions. |
| Household size | 7 | Person responsible for purchasing eggs, consumes eggs at dinner/evening meal, preferred purchase format, preferred purchase location, importance of refrigeration, knows animal welfare concept, uses product origin as a quality cue. |
| Place of residence | 11 | Person responsible for purchasing eggs, preferred shell color, preferred purchase location, uses product origin as a quality cue, associates yolk color with a fresher egg, associates yolk color with production system, importance of cholesterol content, importance of omega-3 and omega-6 content, importance of protein content, importance of vitamin content, importance of low carbon footprint certification. |

***Note.*** *Only key outcomes with significant associations are shown.*
